# Enhancing Wastewater Treatment with Aerobic Granular Sludge: Impacts of Tetracycline Pressure on Microbial Dynamics and Structural Stability

**DOI:** 10.3390/microorganisms12091913

**Published:** 2024-09-20

**Authors:** Shengyan Zheng, Bichen Lou, Zhonghui Yang, Dong Ou, Ning Ai

**Affiliations:** 1 College of Biological, Chemical Science and Engineering, Jiaxing University, Jiaxing 314001, China; m18268301932@163.com (S.Z.); loubichen0130@163.com (B.L.); 221122010316@zjut.edu.cn (Z.Y.); 2 College of Chemical Engineering, Zhejiang University of Technology, Hangzhou 310014, China

**Keywords:** aerobic granular sludge, tetracycline, extracellular polymeric substances, wastewater treatment, microbial community

## Abstract

This study evaluated the efficiency of aerobic granular sludge (AGS) technology in treating wastewater contaminated with tetracycline (TC), a common antibiotic. AGS was cultivated under a TC pressure gradient ranging from 5 mg/L to 15 mg/L and compared with conventional wastewater conditions. The results demonstrated that AGS achieved high removal efficiencies and exhibited robust sedimentation performance, with significant differences in average particle sizes observed under both conditions (618.6 μm in TC conditions vs. 456.4 μm in conventional conditions). Importantly, exposure to TC was found to alter the composition and production of extracellular polymeric substances (EPSs), thereby enhancing the structural integrity and functional stability of the AGS. Additionally, the selective pressure of TC induced shifts in the microbial community composition; *Rhodanobacter* played a crucial role in EPS production and biological aggregation, enhancing the structural integrity and metabolic stability of AGS, while *Candida tropicalis* demonstrated remarkable resilience and efficiency in nutrient removal under stressful environmental conditions. These findings underscore the potential of AGS technology as a promising solution for advancing wastewater treatment methods, thus contributing to environmental protection and sustainability amid growing concerns over antibiotic contamination.

## 1. Introduction

Tetracycline (TC) represents one of the most widely utilized classes of antibiotics globally, celebrated for their broad-spectrum antimicrobial properties, efficacy, and cost-effectiveness [1]. These antibiotics found extensive usage across human healthcare, veterinary practices, and agriculture sectors. A notable quantity of tetracycline, along with other antibiotics, is excreted unmetabolized by both humans and animals, leading to their continuous accumulation in the environment [1,2]. Thus, TC was often detected in various types of wastewaters with concentrations ranging from the level in ng/L to mg/L [2]. The accumulation of TC in different environmental media posed risks to non-target organisms and facilitated the development of antibiotic resistance [3,4]. This situation highlighted the critical necessity for improving wastewater treatment methods to minimize the environmental discharge of tetracyclines.

Meanwhile, the conventional sewage treatment technology was not designed to remove antibiotics [3,5]. Traditional activated sludge systems are widely used for wastewater treatment but often struggle with antibiotics, including TC, due to their persistence and low biodegradability. These systems may fail to completely remove or degrade antibiotics, leading to environmental concerns. Furthermore, the presence of antibiotics in wastewater could severely impair the biological processes essential for wastewater treatment by inhibiting the activity of crucial microbial populations, resulting in reduced treatment efficiency and the buildup of harmful byproducts in the effluent [6]. Aerobic granular sludge (AGS) technology presented a compelling alternative for the treatment of antibiotic-contaminated wastewaters, which were characterized by superior settling properties, reduced sludge production, and enhanced resilience to toxic compounds relative to conventional activated sludge processes [7,8,9,10,11,12,13,14]. AGS systems demonstrated an increased capacity for handling elevated contaminant loads and offer improved operational stability. Consequently, AGS technology held significant promise in addressing the limitations associated with existing wastewater treatment methods. Furthermore, the robust structure of AGS, the unique three-dimensional structure, acted as a buffer against TC exposure, resulting in the great application prospect for treating TC pharmaceutical wastewater [15]. In addition to its superior settling characteristics, the robust structure of AGS offered distinct advantages for treating wastewater contaminated with TC [15]. The unique three-dimensional architecture of AGS provided a structural buffer that enhanced the resilience of AGS system to the adverse effects of TC exposure. This structural integrity helped mitigate the impact of TC on microbial activity and overall system performance. As a result, AGS technology demonstrated significant potential for effectively managing and treating TC-containing pharmaceutical wastewater. The resilience and adaptability of AGS systems in the face of such contaminants underscored their promising application prospects in advanced wastewater treatment scenarios.

Extracellular polymeric substances (EPSs), synthesized by microorganisms, were critical components that governed the physicochemical and biological characteristics of biomass in wastewater treatment systems [16,17]. The EPS matrix was identified as serving dual roles: acting as protective net buffer layers to shield cells from external shocks and functioning as a conduit among cells, thus enhancing the flocculation of sludge [18,19]. Consequently, EPS was deemed the most crucial factor in the granulation, stability, and performance of AGS [16]. Studies have shown that the presence of TC could lead to notable changes in the composition and production of EPS, which may impact the treatment efficiency and resilience of microbial communities [6]. Thus, further research on the dynamic relationship between EPS components and content, removal efficiency, and microbial community succession under TC exposure conditions was crucial for developing effective wastewater treatment strategies and coping with environmental challenges caused by antibiotic pollution.

Therefore, this study aimed to rigorously investigate the cultivation of AGS under the selective pressure of TC, with a focus on elucidating its physiological, biochemical, and biodegradation characteristics, as well as the kinetics, pathways, and mechanisms involved. Additionally, this research explored the dynamic interplay between EPS and microbial communities in the context of TC exposure, assessing how such exposure influences EPS composition and production, treatment efficiency, and microbial succession. The findings of this study were anticipated to contribute significantly to the development of effective strategies for the treatment of antibiotic-contaminated wastewater and to address the pressing environmental challenges associated with antibiotic pollution.

## 2. Materials and Methods

### 2.1. Experimental Setup and Operating Conditions

Two AGS reactors, R1 and R2, were operated under controlled conditions over a 42-day period. R1 served as a control, while R2 was exposed to varying concentrations of TC. Each reactor comprised a cylindrical glass vessel measuring 50 cm in height, with an inner diameter of 6 cm and a working height of 40 cm. The aspect ratio of 8 yielded a volume of 1 L. Inlet and outlet operations were regulated by peristaltic pumps, while aeration was facilitated through a bottom-mounted diffuser driven by an aeration pump. Automated control of the SBR system was achieved via a programmable time controller. Each reactor completed four cycles daily, each lasting 6 h, with a volume exchange ratio of 50% to ensure adequate replenishment of reactor contents. Aeration was maintained at a constant rate of 3 L/min to facilitate oxygen transfer and promote reactor mixing. Rigorous temperature control was enforced within a range of 22 ± 2 °C throughout the 42-day operational period. Simultaneous optimization of operational parameters for R1 and R2 reactors was undertaken to bolster reactor efficiency and foster aerobic granular sludge formation. Table 1 presented a comprehensive overview of the specific operating parameters employed during the experimental endeavor.

### 2.2. Seed Sludge and Wastewater Composition

The seed sludge was obtained from the recovered sludge of the secondary clarifier at the Jiaxing municipal wastewater treatment plant in China [15]. The simulated wastewater utilized in this investigation was formulated to closely mimic real-world conditions. The principal constituents, quantified in milligrams per liter (mg/L), were as follows: C_6_H_12_O_6_ at 937 mg/L, NH_4_Cl at 500 mg/L, KH_2_PO_4_ at 140 mg/L, CaCl_2_ at 150 mg/L, MgCl_2_ at 31 mg/L, and FeSO_4_·H_2_O at 10 mg/L. The pH was adjusted to a target range of 7.0 ± 0.1 using either 2M NaHCO_3_ or HCl. The calculated C/N/P ratio was approximately 11.8:4.1:1.

To ensure the presence of essential trace elements, a supplementary trace element solution was prepared and added to the simulated wastewater at a ratio of 2.5 mL per 25 L. The solution comprised the following components, each quantified in mg/L: H_3_BO_3_ at 50 mg/L, ZnCl_2_ at 50 mg/L, CuCl_2_ at 30 mg/L, MnSO_4_·H_2_O at 50 mg/L, (NH_4_)_6_Mo_7_O_24_·H_2_O at 50 mg/L, AICl_3_ at 50 mg/L, CoCl_2_·6H_2_O at 50 mg/L, and NiCl_2_ at 50 mg/L [20].

To simulate conditions of TC contamination within the R2, TC was incrementally introduced into 25 L of the simulated wastewater at predefined concentrations over specific time intervals. The concentrations of TC were systematically maintained at 5 mg/L for the initial period of 0–14 days, elevated to 10 mg/L for the subsequent period of 15–28 days, and further increased to 15 mg/L for the final period of 29–42 days.

### 2.3. Analysis of Conventional Sludge Indicators

Conventional sludge indicators including sludge volume index (SVI), mixed liquor suspended solids (MLSSs), mixed liquor volatile suspended solids (MLVSSs), and chemical oxygen demand (COD) were measured using standard methods [21]. TC concentration was determined by high-performance liquid chromatography (HPLC, Shimadzu, Kyoto, Japan), and detailed test conditions were depicted by Zhang et al. [2]. Specific oxygen uptake rate (SOUR) was measured following established methods [22], using a dissolved oxygen meter to track changes in dissolved oxygen levels over time. The integrity coefficient (IC) measurement method involved determining the ratio of residual particles to the total weight of granular sludge [23], achieved by subjecting the sludge to mechanical agitation at 200 rpm for 5 min using a vortex oscillator. The particle size distribution of the sludge at each stage was monitored by Malvern particle size analyzer (Mastersizer 2000, Malvern, UK) [24].

### 2.4. EPS Extraction and Analysis

EPS extraction from aerobic granular sludge samples was conducted through heat extraction, aligning with prior investigations [25]. First, 40 mL sludge-water samples were triple-rinsed with deionized water in 50 mL centrifuge tubes through low-speed centrifuge. Ultrasonication was performed at 21 kHz for 5 min using a Branson 2510 ultrasonic cleaner (Danbury, CT, USA). Samples were then agitated horizontally at 150 rpm for 20 min. Following this, samples were centrifuged at 8000 rpm for 10 min. The supernatant was removed, and the sludge was resuspended in deionized water. The mixture was heated at 60 °C for 30 min and centrifuged again at 8000 rpm. The supernatant was filtered through a 0.25 μm polycarbonate filter membrane using a syringe filter unit. EPS retention was controlled by verifying filter efficiency with known EPS concentrations and ensuring minimal loss.

#### 2.4.1. Quantitative Analysis of EPS

Quantification of polysaccharides (PSs) and proteins (PNs) within the extracted EPS from sludge samples under diverse cultivation conditions was undertaken. PS content was determined utilizing the phenol–sulfuric acid method with glucose as standard at 490 nm [26], while PN content was assessed employing the BCA method with bovine serum albumin as standard at 562 nm [24].

#### 2.4.2. Qualitative Analysis of EPS

Qualitative analysis of EPS was conducted through three-dimensional excitation emission matrix fluorescence spectroscopy (3D-EEM) (Hitachi F-4700, Beijing, China), enabling the elucidation of fluorescent substance excitation and emission wavelengths, with fluorescence intensity varying accordingly [27]. Spectral data were collected with excitation and emission wavelengths set at 200–450 nm and 250–500 nm, respectively, using a 10 nm excitation and emission slit width and a scanning speed of 12,000 nm/min. Blank controls comprised deionized water samples, and spectral data were processed using Origin (Pro2021).

### 2.5. Metagenomic Sequence, Assembly and Analysis

Samples of granular sludge were collected at 0 and 42 days, designated as S1 (seed sludge), S2 (mature sludge from R1 at day 42), and S3 (mature sludge from R2 at day 42). Each sample included three biological replicates to ensure statistical reliability. Microbial genomic DNA was extracted using the OMEGA Soil DNA Kit (D5625-01, Shanghai, China) in accordance with the manufacturer’s protocol and subsequently stored at −20 °C. The quality and quantity of the extracted DNA were assessed via agarose gel electrophoresis and a NanoDrop spectrophotometer (ND-1000 Fisher Scientific, Waltham, MA, USA). Metagenome shotgun sequencing libraries with 400 bp insert sizes were constructed utilizing the Illumina TruSeq Nano DNA LT Library Preparation Kit (Illumina, San Diego, CA, USA). Sequencing was performed on a Novaseq 6000 platform (Illumina, San Diego, CA, USA) by Personal Biotechnology Co., Ltd. (Shanghai, China). Rigorous quality control measures were applied to the sequencing data to eliminate contamination and ensure the generation of high-quality datasets suitable for downstream metagenomic analysis. 

## 3. Results and Discussion

### 3.1. Effect of TC on AGS Formation and Performance

#### 3.1.1. Morphological Evolution of Aerobic Granular Sludge

**Figure 1:**
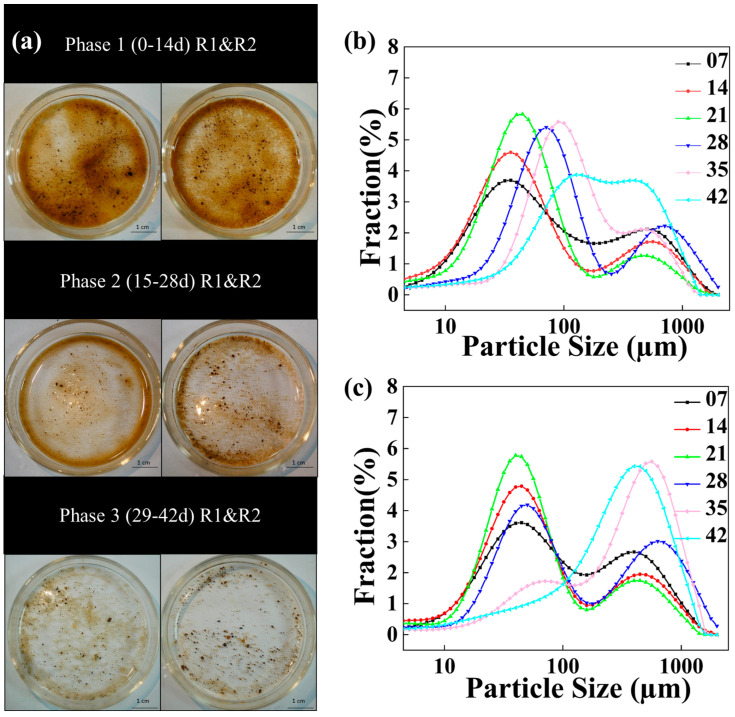
Morphology and particle size succession of sludge in granulation: (**a**) morphology of sludge; (**b**) particle size succession of R1; (**c**) particle size succession of R2.

**Figure 2:**
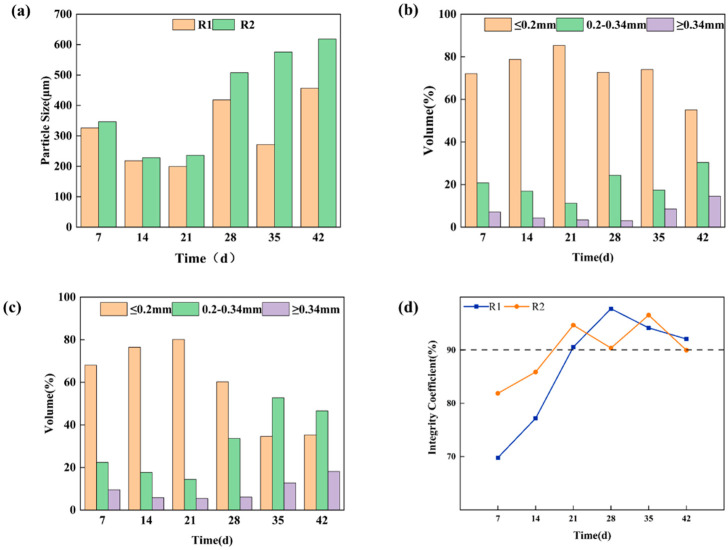
Physical properties during granulation process: (**a**) average particle size; (**b**) particle size distribution of R1; (**c**) particle size distribution of R2; (**d**) IC.

The formation process of AGS was described in this study, examining both macro and micro perspectives. As shown in Figure 1 Phase 1, the sludge concentration was relatively higher in both R1 and R2 compared to Phase 2 and Phase 3, with a loosely structured sludge matrix. Initially, the majority of the sludge in both reactors remained fragmented, with an average particle size ranging from 200 to 350 μm (Figure 2a). Additionally, during the initial stages of AGS formation, over 60% of the sludge had an average particle size ≤ 0.2 mm (Figure 2c).

During the cultivation stage of 15–28 d (Figure 1a Phase 2), there was a pronounced enhancement in sludge agglomeration in both R1 and R2, resulting in an increase in the average particle size. In detail, the average particle size of R1 increased from 199.4 μm to 418.4 μm, while that of R2 increased from 236.2 μm to 507.6 μm. Additionally, there was a significant increase in the proportion of sludge with particle sizes ranging from 0.2 to 0.34 mm, with the proportion rising from 11.2% to 24.3% in R1 and from 14.4% to 33.6% in R2, respectively (Figure 2c).

During the cultivation period of 29–42 d (Figure 1a Phase 3), the degree of sludge granulation further increased. For R1, the average particle size increased from 271.4 μm to 456.4 μm, with 55.1% of sludge particles exhibiting a size of ≤0.2 mm, 33.4% in the size range of 0.2–0.34 mm, and 14.5% with the size of ≥0.34 mm on day 42 (Figure 2c). For R2 with TC addition, the average particle size of sludge illustrated an increasing trend from 236.2 μm to 618 μm in 21–42 d, accompanied by significant changes in particle size distribution. In detail, the proportion of sludge particle size of ≤0.2mm gradually decreased from 80.1% to 35.3%, while the proportion of sludge particle size of 0.2–0.34 mm gradually increased from 14.4% to 46.6% in 21–42 d. Compared with R1, the AGS in R2 had a larger particle size. Ou et al. [17] reported that the increase in particle size of granular sludge was beneficial for protecting internal microorganisms from adverse external environments. In this study, the enlargement of granule diameter maximized the protection of internal microorganisms from the influence of tetracycline.

According to previous research, IC can serve as a reliable indicator for assessing the maturity of granular sludge when IC > 90% [28]. In this experiment, the cultivated granular sludge exhibited a continuous increase in IC during the 0–21 d stage (Figure 2d). At 21 d, the IC of R1 was 90.6%, and IC of R2 was 94.7%, indicating that both R1 and R2 achieved sludge maturation. Throughout the 22–42 d stage, the IC values remained above 90% in both R1 and R2. Specifically, during the 22–28 d stage, R1 demonstrated an IC of 97.8%, whereas R2 exhibited an IC of 90.4% (Figure 2d). Similarly, in the subsequent stages (29–35 d and 36–42 d), R1 displayed respective ICs of 94.2% and 92.1%, while R2 showcased corresponding values of 96.6% and 90%. Observations of sludge morphology further substantiated these findings, with distinct sludge particles observed around the 20-day mark, signifying successful granulation and robust particle strength in the cultivated granular sludge from both reactors. The results confirmed that clear sludge particles were visible after approximately 20 days, and the addition of TC facilitated the maturation process of AGS. The results had significant implications for the pharmaceutical wastewater containing TC treatment process, suggesting that maintaining a lower concentration of TC promoted the development and stability of AGS, thus enhancing the efficiency and sustainability of biological wastewater treatment systems.

#### 3.1.2. Performance of Aerobic Granular Sludge

##### Sludge Concentration

**Figure 3:**
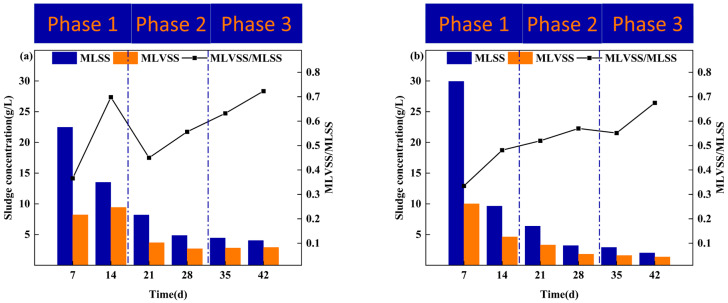
Sludge concentration in granulation: (**a**) R1; (**b**) R2.

The performance of AGS in terms of sludge concentration was evaluated over a 42 d cultivation period in R1 and R2. As illustrated in Figure 3, both reactors exhibited a similar trend of sludge concentration, characterized by an initial decrease followed by an increase. Notably, MLSSs in R1 consistently exceeded that of R2 throughout the cultivation process.

During the 0–14 d stage, MLSSs in R1 decreased from an initial value of 22.47 g/L to 13.51 g/L, while MLSSs in R2 decreased from 29.95 g/L to 9.64 g/L. In the subsequent 15–21 d stage, the settling time was reduced to 15 min, leading to the exclusion of poorly settling sludge from the reactor, thereby favoring the development of the good settling performance of AGS. And it resulted in a decrease in MLSSs to 8.20 g/L for R1 and 6.39 g/L for R2. As the cultivation progressed to the 29–35 d stage, with a settling time of 5 min and increased hydraulic selective pressure, MLSSs values decreased even further, reaching the lowest values of 4.46 g/L for R1 and 1.22 g/L for R2. In the 36–42 d stage, MLSSs increased slightly to 4.04 g/L for R1 and 2.03 g/L for R2, indicating the maturation of aerobic granular sludge and the retention of sludge with good settling performance in the reactor.

##### Removal Performance

**Figure 4:**
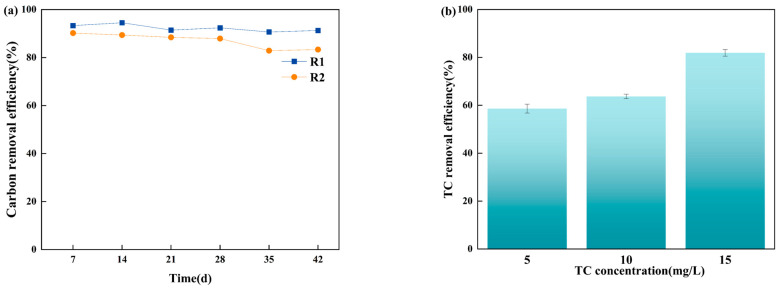
Removal performance for AGS: (**a**) carbon removal efficiency (%) for R1 and R2; (**b**) TC removal efficiency (%) for R2.

The performance of the AGS system was evaluated in terms of carbon and TC removal efficiencies over a 42-day cultivation period, as shown in Figure 4. Both reactors displayed similar trends in carbon removal efficiency (Figure 4a). R1 achieved a higher overall removal efficiency, maintaining approximately 90%, compared to R2, which sustained over 82%. Initially, the addition of TC disrupted the AGS system by inhibiting the activity of certain microbial populations crucial for the degradation processes. The disruption likely caused a temporary decline in AGS performance. However, as AGS formation and maturation progressed, the negative impact of TC diminished. The results were consistent with our previous study [15], which observed a similar pattern of initial inhibition followed by recovery and adaptation in AGS systems exposed to TC. In terms of TC removal (Figure 4b), the performance of R2 was monitored throughout the 42-day period. The TC removal efficiency by the AGS system increased with higher initial TC concentrations. Specifically, with initial TC concentrations of 5 mg/L, 10 mg/L, and 15 mg/L, the average degradation efficiencies were 58.62%, 63.72%, and 81.91%, respectively. With the cultivation of AGS, the microorganisms in the AGS system gradually adapted to the TC environment, resulting in a steady increase in the TC removal rate. On day 42, the TC removal rate exceeded 80%, demonstrating a stable upward trend throughout the operation of R2.

##### Sludge Activity

**Figure 5:**
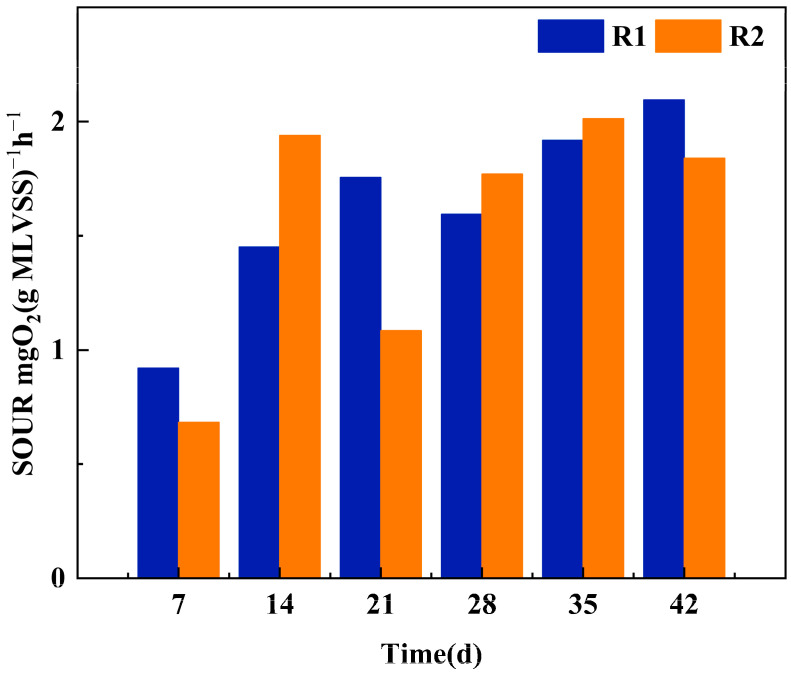
The SOUR of AGS under different culture conditions.

The sludge biological activity during the formation of AGS was characterized by SOUR, as shown in Figure 5. During the incubation period of 0–14 d and the 30 min settling time under operating conditions, the SOUR of R1 increased from 0.336 mg O_2_ (g MLSSs)^−1^h^−1^ to 1.013 mg O_2_ (g MLSSs)^−1^h^−1^, and SOUR of R2 was slightly lower than that of R1 due to TC addition. The SOUR of R1 also increased from 0.228 mg O_2_ (g MLSSs)^−1^h^−1^ to 0.934 mg O_2_ (g MLSSs)^−1^h^−1^. For 15–28 days during the cultivation stage, with the gradual formation of aerobic granular sludge, the overall activity improved. During this stage, the SOUR of R1 increased from 0.790 mg O_2_ (g MLSSs)^−1^h^−1^ to 0.887 mg O_2_ (g MLSSs)^−1^h^−1^, and the SOUR of R2 increased from 0.563 mg O_2_ (g MLSSs)^−1^h^−1^ to 1.009 mg O_2_ (g MLSSs)^−1^h^−1^. For 29–42 days during the cultivation stage, the granular sludge gradually matured, and the SOUR continued to be higher than 1.000 mg O_2_ (g MLSSs)^−1^h^−1^. When the sludge activity is higher than the initial stage of cultivation by 35 days, the SOUR of R1 is 1.212 mg O_2_ (g MLSSs)^−1^h^−1^, and the SOUR of R2 is 1.515 mg O_2_ (g MLSSs)^−1^h^−1^; at 42 d, the SOUR of R1 is 2.656 mg O_2_ (g MLSSs)^−1^h^−1^, and the SOUR of R2 is 1.241 mg O_2_ (g MLSSs)^−1^h^−1^. As antibiotics inhibit cell activity, overall, the biological activity of sludge in R2 is slightly lower than that of sludge in R1.

### 3.2. Effect of TC on EPS Characteristics of Aerobic Granular Sludge

#### 3.2.1. Quantitative Analysis of EPS of Aerobic Granular Sludge

**Figure 6:**
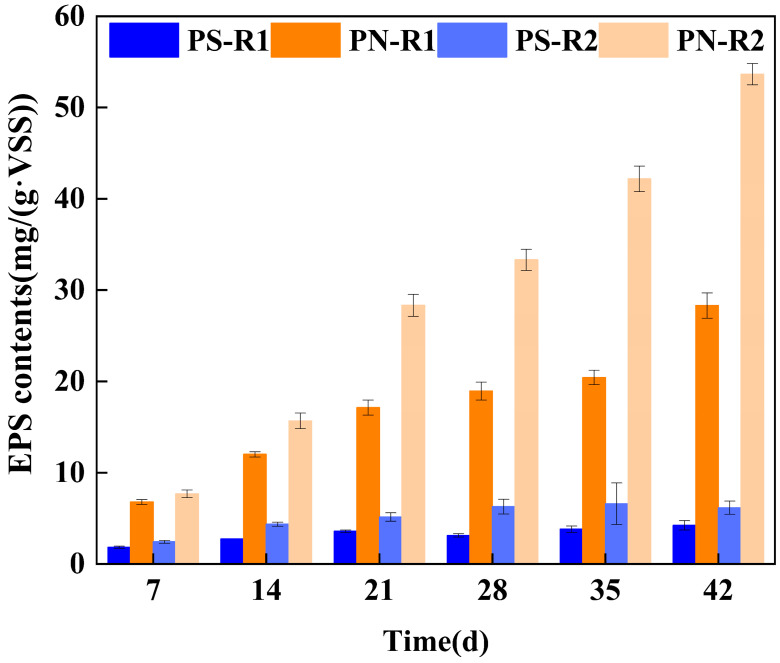
EPS contents at different culture conditions: PN and PS contents in EPS in R1 and R2.

PN and PS were employed as the main components of EPS to investigate the variation in EPS content with TC exposure. As shown in Figure 6, the concentration of PN and PS was all gradually increased both in R1 and R2, and the increase in PN concentration was greater than that of PS concentration. During the incubation period of 0–14 d, the PN increased from 6.782 ± 0.27 mg/(g·VSS) to 12.014 ± 0.284 mg/(g·VSS) in R1, while the PN increased from 7.685 ± 0.43 mg/(g·VSS) to 15.679 ± 0.87 mg/(g·VSS). And with the cultivation of AGS, the PN content showed a continuous increasing trend both in R1 and R2. On day 42, the PN content in R1 was 28.3 ± 1.382 mg/(g·VSS), while in R2 it was 53.662 ± 1.382 mg/(g·VSS), approximately twice that of R1. Compared with PN concentration, the variation in PS concentration was not significant. In R1, the PS concentration increased from 1.832 ± 0.122 mg/(g·VSS) to 4.237 ± 0.514 mg/(g·VSS) over 0–42 d, and in R2, it increased from 2.422 ± 0.15 mg/(g·VSS) to 6.166 ± 0.73 mg/(g·VSS) over 0–42 d. The increase in PN content observed in both R1 and R2 signified an enhancement in the settling performance of the AGS system within these two reactors. Zhang et al. [29]. found a strong correlation between the increase in PN content and good sludge settling performance. Comparing the PN content under different culture conditions, it was observed that the PN content in R2 was higher than that in R1 during 0–42 days. This could be attributed to the infiltration of TC into the cells, leading to the secretion of more extracellular PN to protect the cells from the toxic effects of TC [30]. TC addition moderately enhanced EPS secretion in AGS, thereby safeguarding the microorganisms within the granular sludge and further augmenting the degradation efficiency of TC in aerobic granular sludge.

#### 3.2.2. Qualitative Analysis of EPS of Aerobic Granular Sludge

**Figure 7:**
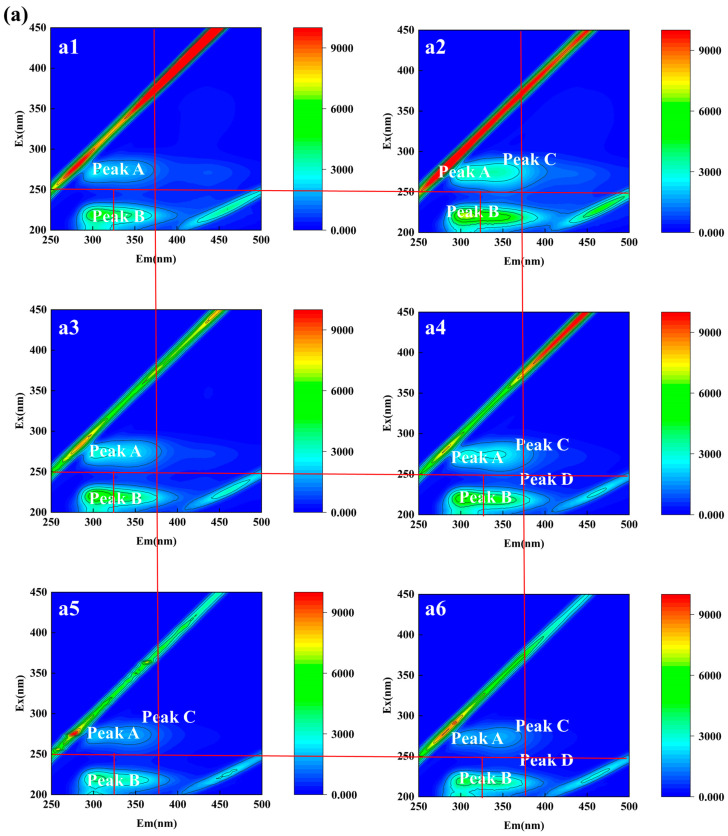

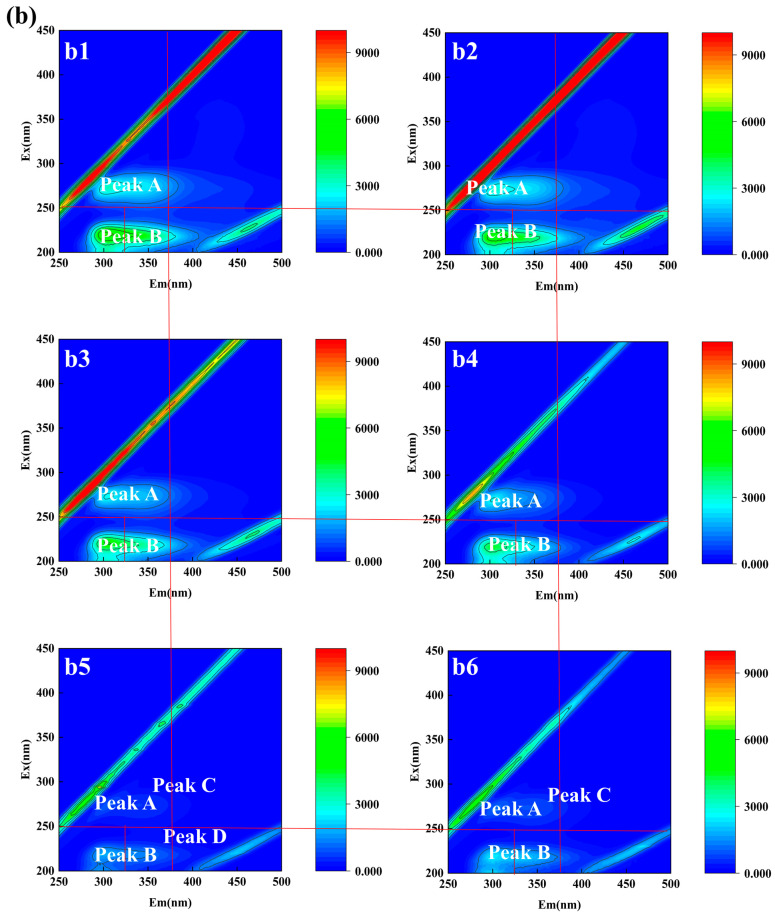
(**a**) EEM fluorescence spectra of EPS in R1: (**a1**) 7 d, (**a2**) 14 d, (**a3**) 21 d, (**a4**) 28 d, (**a5**) 35 d, (**a6**) 42 d; (**b**) EEM fluorescence spectra of EPS in R2: (**b1**) 7 d, (**b2**) 4 d, (**b3**) 21 d, (**b4**) 28 d, (**b5**) 35 d, (**b6**) 42 d.

We employed 3D-EEM to delineate alterations in EPS components under TC exposure in AGS (Figure 7). According to Chen et al. [31], the 3D-EEM spectrum is divided into five regions, with each EEM identifying the special chemical components within EPS samples. In this study, EPS samples extracted under different culture conditions revealed five fluorescence peaks. Peak A and Peak C were identified as tryptophan proteins, while Peak B and Peak D were characterized as aromatic proteins. Additionally, a fluorescence peak of humic acid was observed at Ex/Em of 272/438 (Peak E). With the increase in culture time, the fluorescence intensity of the EEM spectrum peaks of EPS in R1 and R2 showed a gradually decreasing trend overall (Table 2). Comparative analysis of EEM spectra revealed that under TC pressure, the fluorescence intensity of EPS peaks was notably reduced compared to normal conditions. This decrease likely resulted from the inhibitory effect of TC on microbial protein synthesis in AGS. Additionally, humic acids (Peak E) were only observed under normal culture conditions within the first 7 days, which may be due to the growth of bacteria or the decomposition of dead cells and macromolecular organic material [32,33]. These findings aligned with previous research highlighting the impact of antibiotics like TC on microbial growth and metabolic pathways. In R1, under normal culture conditions, EPS fluorescence characteristics appeared to be influenced by microbial growth and activity. Conversely, TC exposure led to alterations in EPS composition, affecting the intensity and nature of fluorescence signals. The observed changes underscored the influence of TC on microbial metabolism and EPS characteristics, supporting the hypothesis that antibiotics could significantly modulate microbial community interactions and EPS properties.

### 3.3. Effect of TC on Microbial Community in Aerobic Granular Sludge

#### 3.3.1. Alpha Diversity Analysis

**Figure 8:**
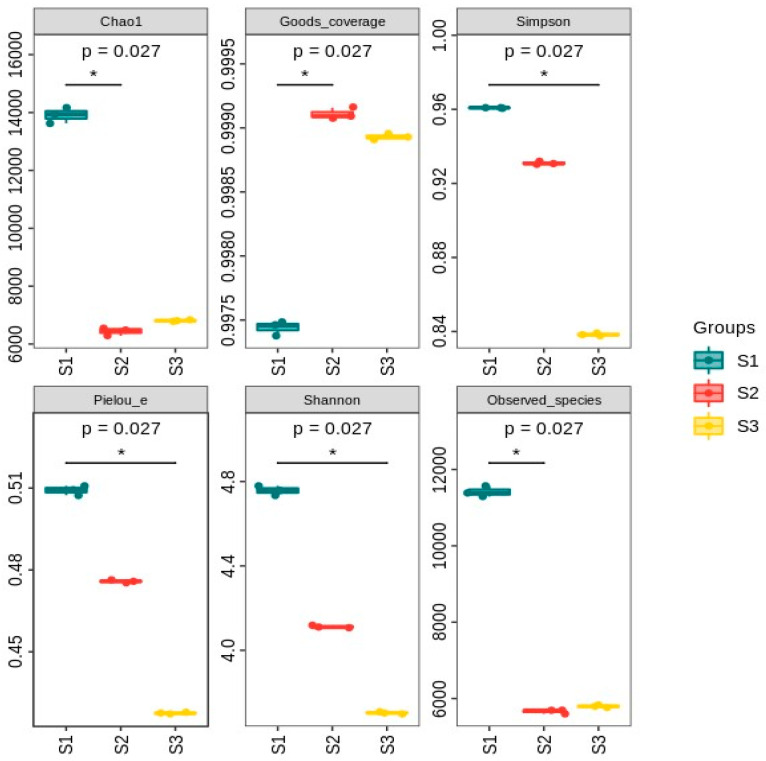
Diversity analysis of species for different sludge samples (S1 stands for seed sludge; S2 stands for mature sludge from R1 at day 42; S3 stands for mature sludge from R2 at day 42; * stands for post-test significant ability marker).

To access the effect of TC on the succession of the microbial community in AGS, metagenomic sequencing analysis was conducted on the seed sludge and mature sludge collected on day 42 from R1 and R2. A total of 22,729,095,237 effective reads were obtained in this study (6,321,767,728 in seed sludge; 8,245,549,237 in mature sludge from R1; 8,161,778,272 in mature sludge from R2). The rarefaction curves displayed stability, indicating they accurately represented the microbial diversity in the sludge samples (Figure 8). Additionally, the Goods coverage index was 99.74%, 99.91%, and 99.89%, respectively, confirming comprehensive coverage of the biological samples.

The Chao1 index, a measure of species richness, was 13,912.36 for seed sludge, 6438.67 for mature sludge from R1, and 6807.57 for mature sludge from R2. These values indicated a reduction in species richness with prolonged cultivation, suggesting an impact of operational conditions on microbial richness [34], and the addition of TC had little effect on microbial richness. Furthermore, the number of observed species decreased from 11,416 at day 0 to 5660 in mature sludge from R1 and 5795 in mature sludge from R2 at day 42, which further supported that the influence of TC on biological community richness was limited. Community diversity was evaluated using the Shannon index and Simpson index [35]. In detail, the Shannon index decreased from 4.757 at day 0 to 4.112 in mature sludge from R1 and 3.704 in mature sludge from R2 at day 42. Similarly, the Simpson index decreased from 0.961 at day 0 to 0.931 in mature sludge from R1 and 0.838 in mature sludge from R2 at day 42. These results indicated that the presence of TC negatively impacted the diversity of the microbial community in AGS.

#### 3.3.2. Structural Analysis of the Microbial Community

**Figure 9:**
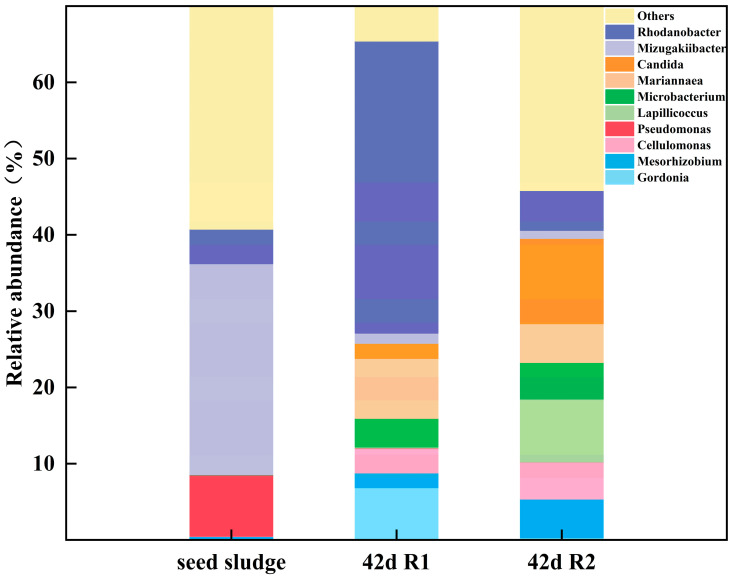
Microbial community of AGS of different sludge samples.

With the aim of exploring the community succession characteristics of AGS with settling time decreasing and antibiotic pressure changing, the microbial community at the species/genus/family/order level was also studied in both the 0 d sludge and the 42 d sludge (R1 and R2). As settling time depressed and antibiotic pressure strengthened, significant changes were observed at the genus level within the microbial community of sludges, with certain genera exhibiting a deducting or increasing trend (Figure 9). In detail, *Mizugakiibacter* (27.65%), *Pseudomonas* (8.04%), and *Rhodanobacter* (4.53%) were the dominant genera in seed sludge. After 42 d of cultivation, the microbial community in mature AGS both in R1 and R2 demonstrated the different migration changes due to the adaptation to different culture conditions.

In R1 without TC exposure, the *Rhodanobacter* relative abundances increased from 4.53% to 38.3% (as 5.22% in R2) and became the most abundant genus in the R1 sludge system. The genus Rhodanobacter contained a variety of EPS-producing bacteria, which played a key role in promoting biological aggregation and maintaining biological metabolism [36,37]. In this study, *Rhodanobacter* was an indicator of sludge maturation and participated in sludge combination. On the contrary, the *Candida* relative abundances grew from 0.0015% (seed sludge) to 11.19% (R2), while *Candida* in R1 was 1.98%. The *Candida* showed good detoxification performance and had good removal efficiency of nitrogen and phosphorus [38,39]. From the point of view of the dominant or functional microbes, the existence and enrichment of *Candida tropicalis* were the keys to the good performance of AGS under elevated TC pressure. In detail, at the species level, *Candida tropicalis* dominated in mature AGS on d42, and the relative abundance was 12.11%. *Candida tropicalis* has complex cell walls and pseudohyphal and hyphal forms, known as dimorphism, giving them the ability to survive in extreme environments [38,40]. Furthermore, *Candida tropicalis* was found to have excellent nutrient removal performance in wastewater treatment and provided a novel perspective on dimorphic fungi to improve polluted water quality [38], and the addition of *Candida tropicalis* prompted effectively simultaneous removal of carbon, nitrogen, and phosphorus in the activated sludge reactor [40].

## 4. Conclusions

The cultivation of AGS under a TC pressure gradient (5 mg/L to 15 mg/L) and in conventional wastewater conditions demonstrated significant removal efficiencies and robust sedimentation performance, with average particle sizes of 618.6 μm and 456.4 μm, respectively. TC exposure crucially altered the EPS composition and production, enhancing the structural integrity and functional stability of AGS. Furthermore, the selective pressure of TC induced shifts in the microbial community, enriching strains capable of tolerating or degrading the antibiotic. In summary, the cultivation of AGS under TC pressure offers promising avenues for advancing wastewater treatment technologies, ensuring environmental protection and sustainability in the face of increasing antibiotic contamination.

## Figures and Tables

**Table 1:** Operating parameters of SBR reactor.

Time (d)	Feeding (min/Cycle)	Anoxic Time (min/Cycle)	Aerobic Time (min/Cycle)	Settling Time (min/Cycle)	Discharge (min/Cycle)
0–7	2	30	296	30	2
8–14	2	30	296	30	2
15–21	2	30	311	15	2
22–28	2	30	311	15	2
29–35	2	30	316	10	2
36–42	2	30	316	10	2

**Table 2:** Fluorescence spectral parameters of EPS at different culture conditions.

Samples	Time	Peak A		Peak B		Peak C		Peak D		Peak E	
	(d)	Ex/Em	Intensity	Ex/Em	Intensity	Ex/Em	Intensity	Ex/Em	Intensity	Ex/Em	Intensity
R1	0–7	272/304	2572	220/306	5040	-	-	-	-	272/438	849.9
	8–14	274/306	3677	220/310	7218	274/338	3378	-	-	-	-
	15–21	272/306	3095	220/302	6647	-	-	-	-	-	-
	22–28	274/306	2991	220/302	6353	274/342	2649	220/334	5013	-	-
	29–35	274/306	2041	220/302	4099	274/340	1707	-	-	-	-
	36–42	274/306	1865	220/304	4431	274/344	1776	218/334	3925	-	-
R2	0–7	274/306	3210	220/306	6460	-	-	-	-	-	-
	8–14	274/306	3098	220/306	5826	-	-	-	-	-	-
	15–21	274/306	2663	220/306	5082	-	-	-	-	-	-
	22–28	272/304	2578	218/304	4580	-	-	-	-	-	-
	29–35	272/302	953.2	220/300	1795	274/342	630.8	218/330	1496	-	-
	36–42	274/304	1127	218/302	2449	274/336	853.3	-	-	-	-

## Data Availability

The original contributions presented in the study are included in the article, further inquiries can be directed to the corresponding authors.

## Abbreviations

AGS, aerobic granular sludge; TC, tetracycline; EPS, extracellular polymeric substance; SVI, sludge volume index; MLSSs, mixed liquor suspended solids; MLVSSs, mixed liquor volatile suspended solids; COD, chemical oxygen demand; SOUR, specific oxygen uptake rate; PS, polysaccharides; PNs, proteins; 3D-EEM, three-dimensional excitation emission matrix fluorescence spectroscopy; IC, integrity coefficient.
